# The impact of solute carrier proteins on disrupting substance regulation in metabolic disorders: insights and clinical applications

**DOI:** 10.3389/fphar.2024.1510080

**Published:** 2025-01-09

**Authors:** Jiangxia Du, Minhui Shen, Jiajia Chen, Hao Yan, Zhifei Xu, Xiaochun Yang, Bo Yang, Peihua Luo, Kefeng Ding, Yuhuai Hu, Qiaojun He

**Affiliations:** ^1^ Center for Medical Research and Innovation in Digestive System Tumors, Ministry of Education, The Second Affiliated Hospital, Zhejiang University School of Medicine, Hangzhou, Zhejiang, China; ^2^ Center for Drug Safety Evaluation and Research of Zhejiang University, College of Pharmaceutical Sciences, Zhejiang University, Hangzhou, Zhejiang, China; ^3^ Institute of Pharmacology and Toxicology, College of Pharmaceutical Sciences, Zhejiang University, Hangzhou, Zhejiang, China; ^4^ School of Medicine, Hangzhou City University, Hangzhou, Zhejiang, China; ^5^ Department of Pharmaceutical and Translational Toxicology, Innovation Institute for Artificial Intelligence in Medicine of Zhejiang University, Hangzhou, Zhejiang, China; ^6^ Yuhong Pharmaceutical Technology Co., Ltd., Hangzhou, Zhejiang, China

**Keywords:** solute carrier, metabolic disease, transport substrate, transport dysregulation, therapeutic targets

## Abstract

Carbohydrates, lipids, bile acids, various inorganic salt ions and organic acids are the main nutrients or indispensable components of the human body. Dysregulation in the processes of absorption, transport, metabolism, and excretion of these metabolites can lead to the onset of severe metabolic disorders, such as type 2 diabetes, non-alcoholic fatty liver disease, gout and hyperbilirubinemia. As the second largest membrane receptor supergroup, several major families in the solute carrier (SLC) supergroup have been found to play key roles in the transport of substances such as carbohydrates, lipids, urate, bile acids, monocarboxylates and zinc ions. Based on common metabolic dysregulation and related metabolic substances, we explored the relationship between several major families of SLC supergroup and metabolic diseases, providing examples of drugs targeting SLC proteins that have been approved or are currently in clinical/preclinical research as well as SLC-related diagnostic techniques that are in clinical use or under investigation. By highlighting these connections, we aim to provide insights that may contribute to the development of improved treatment strategies and targeted therapies for metabolic disorders.

## 1 Introduction

In recent years, with improvements in people’s living standards and changes in lifestyle, the incidence of metabolic diseases, which have become a major public health problem affecting global economic and social development, is increasing annually worldwide. According to the latest Global Burden of Disease study published in the Lancet in 2020, the greatest risk to health between 1990 and 2019 came from a significant increase in metabolic risk factors (Collaborators, 2020). The metabolic diseases, including type 2 diabetes (T2D), nonalcoholic fatty liver disease (NAFLD), hyperlipidemia, obesity, hyperuricemia, gout, and other related diseases, are associated with dysregulated metabolic processes that involve insulin resistance, the homeostasis of glucose, lipids, and other nutrients, as well as metabolic inflammation (Sunny et al., 2011; Samuel and Shulman, 2016; Xie et al., 2021). These conditions can be either congenital or acquired. Furthermore, increasing understanding underscores the intricate relationship between cancer and metabolic processes, which has led to identifying cancer as a metabolic disorder in which major metabolic pathways are rewired.

The SLC supergroup is the second largest group of membrane proteins, whose members are expressed to varying degrees in different tissues and organs throughout the body and can be distributed in the cell from the nuclear membrane to the plasma membrane of various biological membrane structures. SLC transporters mediate the influx and efflux of ions, amino acids, carbohydrates, neurotransmitters and other endogenous and exogenous substances across biological membranes, which is closely connected to the metabolic equilibrium. Studies have shown that at least 80 SLC proteins are associated with human metabolic diseases, including obesity, T2D, NAFLD, and multiple insulin resistance-related metabolic diseases (Lin et al., 2015a; Schumann et al., 2020), emphasizing the importance of SLC family in regulating normal physiological and pathological functions. However, compared to other membrane protein families, the functions and regulatory mechanisms of SLC membrane proteins are far from well characterized, and only a few members have been identified as drug targets (Schumann et al., 2020). In this review, we will investigate the relationship between SLC families, their corresponding transport substrates, and metabolic diseases, aiming to provide new ideas for the treatment of and targeted drug development against metabolic diseases.

## 2 The SLC proteins

### 2.1 Naming and classification

The SLC gene nomenclature system originated in the 1990s and the naming system begins with “SLC”, followed by “a number representing a family”, then “an English letter (usually A)”, and finally, the number represents “the number of the member in the family” (Hediger et al., 2013). For example, for SLC1A1, this SLC transporter is the first member of the first subfamily of the SLC supergroup.

The Genome Organization Gene Nomenclature Committee (HGNC) mainly classifies SLC proteins based on the different characteristics, homologies, functions and structures of genes, and those with at least 20% sequence identity are considered to constitute the same subfamily (Perland and Fredriksson, 2017). To date, more than 400 SLC proteins have been identified, and have been divided into 65 families based on sequence homology (Pizzagalli et al., 2021), but the exact number of specific proteins remains difficult to determine. Gyimesi et al. identified 120 potential SLC-like proteins using the Transporter Classification Database and Protein families databases (Gyimesi and Hediger, 2022). It is believed that there are many unidentified SLC proteins in the human genome, which need further systematic analysis.

### 2.2 Structural features and functions

To date, the number of three-dimensional structures of eukaryote-derived SLC transporters that have been analyzed is very limited. The analyzed SLC proteins are highly diverse in structure but usually have two common structural characteristics, namely, an asymmetric transmembrane helical structure and a discontinuous helical structure, and their structures often contain multiple folding modes. At present, the two most common folding patterns in the known three-dimensional structure of human SLC proteins are MFS folding and LeuT folding (Bai et al., 2017; Han et al., 2022). The MFS fold usually contains 12 transmembrane helical domains, representing the largest fold cluster in SLC (Colas et al., 2016; Bai et al., 2017), and this fold is found in subfamily proteins, such as SLC2, SLC15, SLC16, SLC17, SLC21, and SLC22 (Doki et al., 2013; Pedersen et al., 2013; Anne and Gasnier, 2014; Holman, 2020). The structural basis of LeuT folding is the 5 + 5 helix structure, which forms two bundles of structures with antiparallel symmetry (Bai et al., 2017) and is found in SLC3, SLC5, SLC6, SLC7 and other subfamily proteins. However, SLC3A1 and SLC3A2 are not transporters, but ancillary proteins involved in the membrane trafficking of other SLC transporters. In addition to the two typical folding patterns of the transporter protein family, there are other transporters with special folding patterns, such as SLC1 with “HP domains”, SLC25 with 6 TMs and 3 similar repeats, and SLC30 with a “V”-shaped homodimer and a C-terminal domain (Bai et al., 2017).

In general, SLC transporters have four main biological functions (Colas et al., 2016): (1) mediating the uptake and transmembrane transport of nutrients or energy materials needed for life activities; (2) participating in the absorption of ions or micronutrients in the body; (3) regulating the transmembrane transport and signaling of neurotransmitters; and (4) working together to transport and efflux drugs, toxins and metabolic wastes. The above biological functions indicate that SLC proteins are involved in the uptake and transport of various metabolites and nutrients within the organism, while metabolic diseases are usually caused by metabolic disorders of related substances in the body, the member names and transport substrates of each SLC family mentioned are presented in Table 1. The following sections will focus on the correlation and regulatory mechanisms between SLC proteins, their transport substrates, and metabolic diseases.

**TABLE 1:** SLC protein family members, their transport substrates, and associated metabolic diseases.

Family	Member	Substrate	Related metabolic diseases
SLC2	GLUT1-12/SLC2A1-12, GLUT13/SLC2A13/HMIT	glucose, fructose, etc. (Raja et al., 2012)	T2D, insulin resistance, NASH, obesity, GLUT1 DS, Fanconi-Bickel Syndrome (Akcan and Silan, 2024)
SLC5	SGLT1, 2/SLC5A1,2	glucose, etc. (Sano et al., 2020)	obesity, diabetes, glucose/galactose malabsorbtion and cardiovascular disease
	SGLT3/SLC5A4	/^a^	diabetes
	SGLT4/SLC5A9	mannose, glucose, fructose, etc. (Tazawa et al., 2005)	diabetes, obesity, diabetic retinopathy
	SGLT5/SLC5A10	mannose, fructose, glucose, galactose (Grempler et al., 2012)	diabetes, chronic nephrosis
	SGLT6/SLC5A11	myo-inositol and D-glucose (Baader-Pagler et al., 2018)	rheumatism (Mathew et al., 2024)
SLC10	NTCP/SLC10A1, ASBT/SLC10A2	bile acid (Kubitz et al., 2014)	sodium-taurocholate cotransporting polypeptide deficiency, hypercholesteremia, NAFLD, diabetes, cholestasis, gall-stone, primary biliary cholangitis (PBC), cholestasis syndrome
	SOAT/SLC10A6	cholesterol, fatty acids (Bhattacharjee et al., 2022)	
	P3/SLC10A3	amino acid	
	P4/SLC10A4, P5/SLC10A5, SLC10A7	/^b^	T2D, osteochondrodysplasia
SLC13	NaS1/SLC13A1, SLC13A4	sulfate, selenate, thiosulfate (Bergeron et al., 2013)	NAFLD, neutrophilic granulocytopenia, diarrhea, hypothioemia
	NaDC1/SLC13A2, NaDC3/SDCT2/SLC13A3, NaCT/SLC13A5	citric acid, α-ketoglutaric acid, succinic acid (Pajor, 2014)	kidney disease, diabetes, epilepsy, obesity, NAFLD
SLC16	MCT1/SC16A1, MCT2/SC16A7, MCT3/SLC16A8, MCT4/SL16A3	L-lactate, pyruvate, short-chain fatty acids, and monocarboxylate drugs, etc. (Felmlee et al., 2020; Bosshart et al., 2021)	lactic acid transport deficiency (Felmlee et al., 2020), monocarboxylate transporter 1 deficiency, diabetic nephropathy, T2D
	MCT5/SLC16A4, MCT11/SLC16A11, MCT13/SLC16A13, MCT14/SLC16A14	/^c^	
	MCT6/SL16A5	xenobiotics (bumetanide, nateglinide, probenecid) (Jones et al., 2017)	
	MCT7/SLC16A6	ketone bodies (Felmlee et al., 2020)	
	MCT8/SLC16A2	thyroid hormones (Thomas et al., 2023)	Allan-Herndon-Dudley syndrome, non-thyroidal illness syndrome (NTIS)
	MCT9/SLC16A9, MCT12/SLC16A12	carnitine (Suhre et al., 2011; Abplanalp et al., 2013)	gout
	MCT10/SLC16A10	aromatic amino acid, thyroid hormones (Halestrap and Meredith, 2004)	inflammatory bowel disease, NTIS, transient neonatal zinc deficiency (TNZD)
SLC17	NPT1/SLC17A1, NPT3/SLC17A2, NPT4/SLC17A3, NPT5/SLC17A4	inorganic phosphate, organic anions (urate, sulfate), glutamate	hyperuricemia, gout and kidney disorders
	SLC17A5	sialic acid, inorganic phosphate	sialic acid storage disease
	VGLUT1/SLC17A7, VGLUT2/SLC17A6, VGLUT3/SLC17A8	glutamate, asparate	obesity, insulin resistance, T2D, NAFLD, hepatic steatosis
	VNUT/SLC17A9	nucleotides (ATP, ADP)	diabetes and metabolic disorders
SLC22	OCT1-3/SLC22A1-3	cationic drugs, carnitine, acetylcholine, dopamine (Nigam, 2018)	
	OCTN1-2/SLC22A4-5	carnitine, acetylcholine, cholin	
	OAT1/SLC22A6	anionic drugs, α-ketoglutarate, urate, indoxyl sulfate	gout, diabetes
	OAT2/SLC22A7	anionic drugs, cGMP, carnitine	renal tubular dysfunction
	OAT3/SLC22A8	anionic drugs, bile acid, carnitine, estrone sulfate	
	OAT7/SLC22A9	estrone sulfate	
	SLC22A10	/^d^	
	OAT4/SLC22A11,OAT10/SLC22A13	urate, estrone sulfate	
	URAT1/SLC22A12	urate	
	SLC22A14-25	estrone sulfate	
SLC25	CIC/SLC25A1	citrate, isocitrate, malate, phosphoenolpyruvate	
	ORC2/SLC25A2	ornithine, citrulline, lysine, arginine, histidine	
	PHC/SLC25A3	phosphate	
	ANT1/SLC25A4 ANT2/SLC25A5 ANT3/SLC25A6	ADP, ATP	
	UCP1/SLC25A7, UCP2/SLC25A8, UCP3/SLC25A9	H^+^(Bouillaud et al., 2016)	obesity, diabetes, hepatitis
	UCP5/BMCP1/SLC25A14	inorganic anions (sulfate, sulfite, thiosulfate and phosphate), dicarboxylates (*e.g.*, malonate, malate and citramalate), aspartate, glutamate and tricarboxylates (Gorgoglione et al., 2019)	
	UCP6/KMCP1/SLC25A30	inorganic anions (sulfate, sulfite, thiosulfate and phosphate), dicarboxylates (*e.g.*, malonate, malate and citramalate), aspartate (Gorgoglione et al., 2019)	
	DIC/SLC25A10	malate, phosphate, succinate, sulphate, thiosulphate	
	OGC/SLC25A11	2-oxoglutarate, malate	
	AGC1/SLC25A12 AGC2/citrin/SLC25A13	glutamate, aspartate (Tavoulari et al., 2022)	steatosis, NASH
	CACT/SLC25A20	carnitine, acylcarnitine (Giangregorio et al., 2017)	
	SCaMC-3/SLC25A23, APC1/SLC25A24, SLC25A25	Mg-ATP/Pi, phosphate and imports adenine nucleotides (Harborne et al., 2017)	fontaine syndrome (Writzl et al., 2017)
SLC27	FATP1-4/SLC27A1-4, FATP6/SLC27A6	LCFA, VLCFA (Pohl et al., 2004; Holloway et al., 2011)	diabetes, NAFLD
	FATP5/SLC27A5	LCFA, bile acids (Kumari et al., 2020; Xu et al., 2023)	
SLC30, SLC39	SLC30s/ZnTs SLC39s/ZIPs	Zn^2+^(Huang and Tepaamorndech, 2013; Thingholm et al., 2020)	diabetes, insulin resistance, TNZD (Lasry et al., 2012)

^a^ SGLT3 acts as a glucose sensor rather than a sugar transporter (Soták et al., 2017).

^b^ SLC10A4 appears to be a protease-activated transporter and transports bile acids; the transport substrates for SLC10A5 and SLC10A7 have not yet been determined (Geyer et al., 2006; Godoy et al., 2007).

^c^ The transport substrates for MCT5, MCT11, MCT13 and MCT14 have not yet been determined (Bosshart et al., 2021).

^d^ SLC22A10 is classified as an orphan transporter with unknown substrates and function (Yee et al., 2023).

## 3 The role of SLC transporters and related substrates in metabolic diseases

Metabolic diseases are a class of disorders caused by dysregulation in metabolic processes within the body, resulting from genetic factors, environmental influences, endocrine imbalances, immune responses, and other factors. Common metabolic diseases can be categorized based on the affected metabolic pathways, including disorders of carbohydrate, lipid, purine metabolism and so on. Next, we will elucidate the role of SLC proteins in metabolic diseases from the perspective of the transport of carbohydrates, bile acids, urate, lipids, monocarboxylates, tricarboxylic acid cycle intermediates, and inorganic ions, as well as the metabolic dysregulation caused by their corresponding SLC transporters.

**FIGURE 1:**
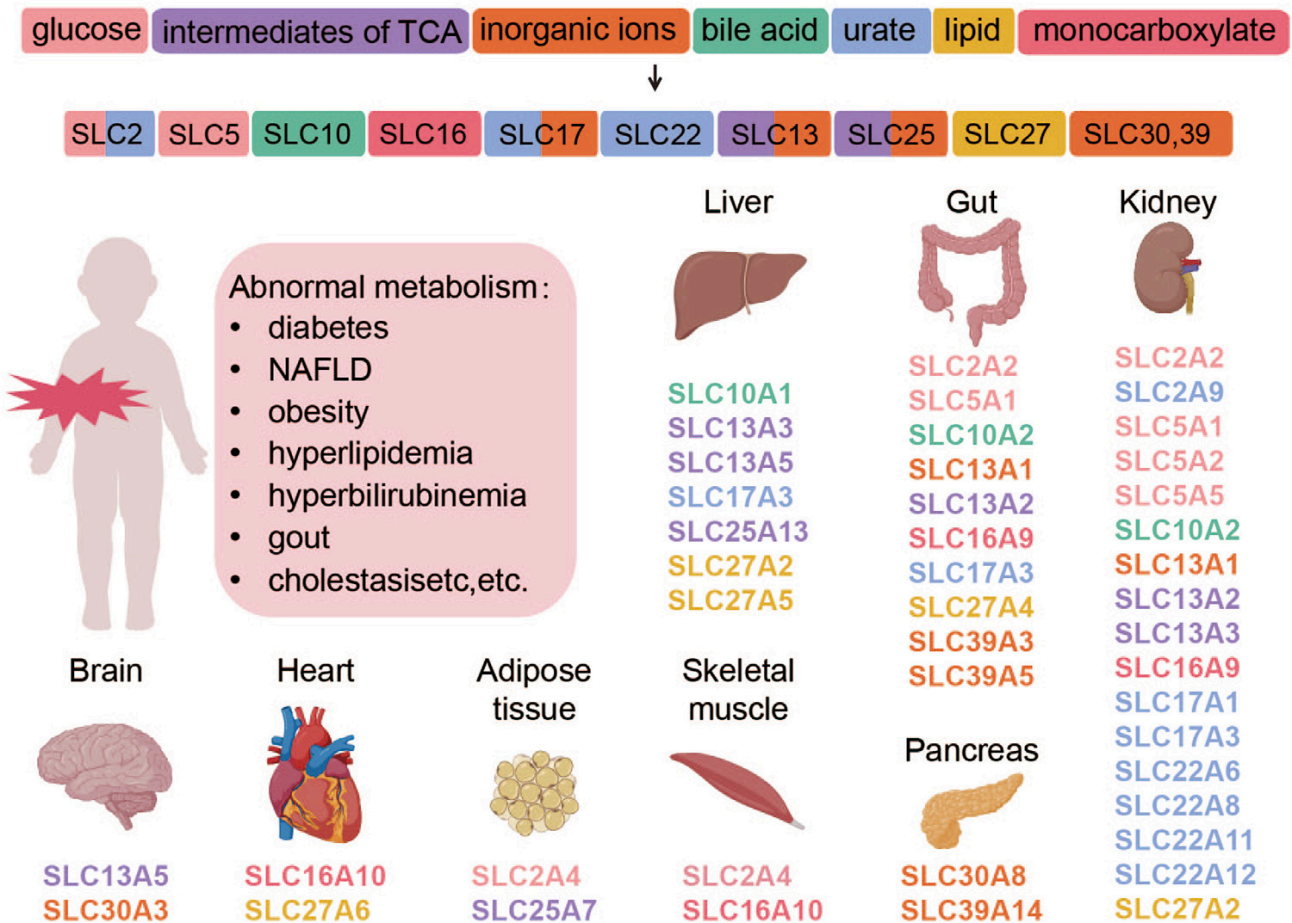
Transporter proteins regulate the movement of various substances, which are categorized by their chemical properties and correspond to specific SLC subtypes. The subtypes, shown with their tissue distributions, are color-coded to match the substance categories. Abnormalities in these transporters are linked to metabolic diseases. This figure was created using BioRender.

The tissue distribution of some SLC protein family members and their corresponding transport substrates are shown in Figure 1.

### 3.1 Carbohydrate transport and metabolism dysregulation

Glucose cannot freely cross the lipid bilayer of the cell membrane, and cellular glucose uptake requires glucose transporters on the cell membrane. Glucose transporters are present in various tissues throughout the body and are classified into two categories: one is sodium-dependent glucose transporter (SGLT) encoded by *SLC5*, which transports glucose against its concentration gradient, and the other is glucose transporter (GLUT) encoded by *SLC2*, which transports glucose along the concentration gradient in the way of facilitated diffusion without consuming energy. Abnormal glucose transport and metabolism can lead to diabetes mellitus, hypoglycemia, hyperglycemia, glycogen storage diseases, impaired glucose tolerance, and insulin resistance.

Apart from classic metabolic diseases, carbohydrate transport dysregulation is also implicated in tumor development. Cancer cells, even when oxygen-rich, still tend to produce energy through aerobic glycolysis, rather than relying on the more efficient mitochondrial oxidative phosphorylation pathway that normal differentiated cells do, a phenomenon termed “the Warburg effect” (Vander Heiden et al., 2009). Due to the presence of the Warburg effect, we have also included the potential relationship between the glucose transporter subfamily of SLC supergroup and tumors.

#### 3.1.1 SLC2 and dysregulation of glucose transport

GLUT encoded by *SLC2* is mainly responsible for the transmembrane transport of glucose in tissues such as liver, skeletal muscle and adipose tissue to ensure a homeostatic balance of blood glucose levels. Thus, most of its members contribute to the development of metabolic diseases such as blood glucose-related T2D and insulin resistance (Chadt and Al-Hasani, 2020). GLUTs facilitate glucose transport through a passive transport mechanism known as facilitated diffusion. Fourteen GLUT protein subtypes have been identified to date and these subtypes can be classified into three subtypes (Mueckler and Thorens, 2013) based on sequence similarity: (1) GLUT1-4 (SLC2A1-4) and 14 (SLC2A14); (2) GLUT5, 7, 9 and 11 (SLC2A5, 7, 9, 11); and (3) GLUT6, 8, 10, 12 (SLC2A6, 8, 10, 12) as well as HMIT (SLC2A13).

GLUT1-4 have been studied in depth. GLUT1 is found in almost every tissue with different levels of expression in different cell types, not including normal liver tissue. And it is expressed in the basolateral membrane. GLUT2 is mainly present in the basolateral membrane of intestine and kidney absorptive epithelial cells (Thorens, 2015), which is also required for glucose sensitive units, particularly in the hepatic portal vein, hypothalamus, and brain stem. GLUT1 and GLUT3 have been proved to be associated with multiple tumor formations and aggressiveness (Ismail and Tanasova, 2022) and have a low correlation with metabolic diseases. Given GLUT2’s involvement in intestinal carbohydrate uptake, it becomes a target of interest for diabetes prevention and treatment by inhibiting intestinal glucose absorption thereby reducing blood glucose levels (Goto et al., 2012).

At the same time, the role of GLUT4 in the regulation of glucose has also attracted much attention. Some existing studies have shown that GLUT4 levels reflect insulin-dependent glucose uptake (Taha et al., 1999). GLUT4 is usually found only in insulin-sensitive skeletal muscle and adipocytes, and decreased *SLC2A4* expression or GLUT4 activity can cause insulin resistance. For example, Guilherme et al. reported that reduced SLC2A4 mRNA and protein levels led to insulin-resistant glucose transport inhibition in adipose tissue in obese or diabetic patients (Guilherme et al., 2008). *Slc2a4* knockout mice have elevated serum glucose and insulin levels, reduced glucose uptake in muscle, and high blood pressure, which are similar to those in T2D (Fam et al., 2012). Mice with homozygous *Slc2a4* gene inactivation are dwarfed, have enlarged hearts, shorter lifespan, and exhibit hyperinsulinemia and insulin resistance in the feeding state (Zisman et al., 2000). The aforementioned research indicates that maintaining GLUT4 protein levels and activity is crucial for glycemic stability. Impairment of GLUT4 translocation is also one of the key factors leading to insulin resistance. Currently, some compounds targeting GLUT4 activity have entered preclinical studies. Details of other preclinical, clinical trial, and marketed drugs targeting SLC can be found in Table 2.

**TABLE 2:** Promising SLC target drugs and the related metabolic diseases.

Targeted SLC members	Metabolic disease	Drugs
GLUT1	GLUT1 deficiency syndrome	Triheptanoin (NCT02014883), Compd 4b (Angeli et al., 2023)
	insulin resistance	Marein (Jiang et al., 2016)
	breast cancer, lung cancer, colon cancer	WZB117 (Zhao et al., 2016), BAY-876 (Guo et al., 2022), STF-31 (Kraus et al., 2018), Glutor^a^(Reckzeh et al., 2019)
	lung fibrosis	Phloretin (Liu et al., 2012)
GLUT4	diabetes	Rhoifolin (Rao et al., 2011) MOTS-c (human) acetate (Lee et al., 2016a) Nepodin (Ha et al., 2014)
GLUT9	gout, hyperuricemia	URAT1/GLUT9-IN-1^a^(Shi et al., 2024)
SGLT1	diabetes	KGA-2727^a^(Shibazaki et al., 2012)
	chronic constipation	Mizagliflozin (NCT02281630)
	diabetic kidney disease	Mizagliflozin
SGLT2	T2D	Canagliflozin (NCT01032629, NCT01989754, NCT02065791), Dapagliflozin (NCT01730534), Ertugliflozin (NCT01986881), Ipragliflozin (UMIN000018440, UMIN000018084), Tofogliflozin (UMIN000017607, UMIN000032601), Luseogliflozin (UMIN000019072, UMIN000021658), Henagliflozin (CTR20131986, CTR20140132) Empagliflozin (NCT01131676)
	hypertension	NCT05090358
	advanced solid tumors	Serabelisib in Combination with Canagliflozin (NCT04073680)
SGLT1, SGLT2	T2D, T1D	Spotagliflozin (NCT02384941, NCT02421510, NCT02531035)
SLC9A3	constipated irritable bowel syndrome, dialysis chronic kidney disease, end-stage renal disease, synucleinopathy-related constipation	Tenapanor (NCT02819687, NCT02796131, NCT06460038, NCT02727751)
MCT1, MCT4	colorectal carcinoma	BAY-8002^a^, AZD3965 (NCT01791595)
VNUT	chronic neuropathic pain	Clodronate^a^
URAT1	gout	Lesinurad (NCT01508702, NCT01510158, NCT01808131, NCT01808144) Dotinurad (NCT02347046, NCT03100318, JPRN-UMIN000054142, NCT06056570) Verinurad (NCT04550234, NCT04532918)
OAT3	hypertension	Thiazide diuretics (NCT02841280, NCT00131846)
NTCP	hepatitis B virus, chronic hepatitis D	Myrcludex B (NCT03852719, NCT03852433, NCT03546621)
ASBT	T2D, primary biliary cholangitis	Linerixibat^a^(Wu et al., 2013) Odevixibat^a^ (Baghdasaryan et al., 2016)
	chronicconstipation, irritable bowel syndrome	Elobixibat (JPRN-jRCTs031200172, JapicCTI-153061 and JapicCTI-153062)
SLC25A4, SLC25A5, SLC25A6	osteoporosis	Clodronate (NCT06263517)
UCP2	T2D	Genipin^a^(Hu et al., 2017)
SLC25A1, GLUT4	NASH, obesity	CTPI-2^a^(Tan et al., 2020)
ZIP7	hepatocellular carcinoma, T cell acute lymphoblastic leukemia	NVS-ZP7-4^a^

^a^ These compounds have not yet entered clinical trials.

Abbreviation: T2D: Type 2 diabetes mellitus; NAFLD: nonalcoholic fatty liver disease; SGLT: Sodium-dependent glucose transporter; GLUT: glucose transporter; FATPs: Fatty acid transport proteins; NASH: nonalcoholic steatohepatitis; ASBT: Sodium-dependent bile acid transporter; NTCP: Na^+^-taurocholate co-transport polypeptide; SOAT: Sodium ion-dependent organic anion transporter; NPT: Na^+^ dependent phosphate transporter; VGLUT: vesicular glutamate transporters; VNUT: vesicular nucleotide transporter; OAT: organic anion transporter; OCT: organic cation transporter; OCTN: organic cation/carnitine transporter; SO4^2-^: inorganic anion sulfate; NAS1: The Na^+^-sulfate cotransporter; SAT1: sulfate anion transporter; NAS: sodium sulfate cotransporters; NADC: Sodium-dependent dicarboxylate transporters; MCT: monocarboxylate transporter protein; ZIP: Zrt-and Irt-like protein family; ZnT: Zinc transporter ZnT family; T1D: Type 1 diabetes mellitus; UCP: uncoupling protein; SDCT: High-affinity sodium-dependent dicarboxylate co-transporter; CACT: Carnitine/acylcarnitine translocase; LC-FAOD: long-chain fatty acid oxidation disorders; IBS: irritable bowel syndrome; PBC: primary biliary cholangitis; NTIS: Non-thyroidal illness syndrome; TNZD: transient neonatal zinc deficiency.

GLUT family-mediated glucose transport is the pacesetter of aerobic glycolysis and, thus, is critical for cancer cell metabolism. Almost all proteins in the SLC2 family have been shown to be associated with the development of cancer (Macheda et al., 2005; Chen and Chen, 2022; Ismail and Tanasova, 2022). GLUT1 upregulation has been observed in cancers such as pancreatic cancer (Li et al., 2023), gastric cancer (Huber and DeRoche, 2023), lung cancer (Zhang et al., 2019), ovarian cancer (Rudlowski et al., 2004), cervical cancer (Rudlowski et al., 2003), and kidney cancer. GLUT3 has been found to be upregulated in various tumors, such as gastric cancer (Yang et al., 2023), colorectal cancer (Dai et al., 2020), and breast cancer (Tsai et al., 2021). Due to its high expression in neurons, GLUT3 is also observed to be upregulated in certain specific tumors, such as neuroblastoma (Ni et al., 2015) and glioblastoma (Libby et al., 2021). GLUT4 and GLUT5 are often found to be upregulated in breast and prostate cancer while GLUT12 is upregulated in human breast tumors. Inhibiting the GLUT12-mediated Warburg effect has been shown to suppress the proliferation, migration, and invasion of breast cancer cells and xenograft tumors (Shi et al., 2020). Other members of the SLC2 family have also been found to be upregulated in various types of cancer; however, research on the mechanisms linking SLC2 to cancer development is still in its early stages.

Current research focuses on inhibiting the function of GLUT proteins, thereby restricting glucose uptake by cancer cells and subsequently inhibiting tumor growth and metastasis (Yadav et al., 2024). These strategies include the use of GLUT small molecule inhibitors (Temre et al., 2022; Chen et al., 2023) and the combination of GLUT inhibitors with chemotherapeutic agents (Weng et al., 2022).

SLC2 family proteins are also implicated in the development of NAFLD, as their transport substrates can either interconvert with lipids or play a role in the physiological regulation of lipid metabolism (Softic et al., 2016). A high-sugar diet, particularly one rich in fructose, is considered a significant contributor to the onset of NAFLD. Unlike glucose, fructose is primarily metabolized in the liver, where excessive intake promotes fat accumulation via the *de novo* lipogenesis pathway. This metabolic process enhances lipid synthesis, making fructose more prone than glucose to induce hepatic fat deposition. Moreover, a high-sugar diet can lead to insulin resistance, a hallmark of NAFLD. Insulin resistance exacerbates hepatic steatosis by further promoting fat accumulation in the liver, thereby contributing to the progression of NAFLD.

The expression levels of several SLC2 family members, including GLUT1, GLUT3, GLUT5, GLUT6, GLUT8, GLUT9, and GLUT12, are significantly upregulated in nonalcoholic steatohepatitis (NASH), a progressive form of NAFLD (Karim et al., 2014). A study on single nucleotide polymorphisms (SNPs) in NAFLD candidate genes revealed 11 SNPs associated with NAFLD, in which 7 were located in the *SLC2A1* gene (Vazquez-Chantada et al., 2013). Further cell experiments demonstrated that silencing *SLC2A1* led to fat accumulation and increased oxidative damage. In addition, studies have shown that GLUT2 and GLUT8 contribute to NAFLD by facilitating fructose uptake (Douard and Ferraris, 2013; DeBosch et al., 2014). Therefore, in the current absence of effective therapeutic strategies for NAFLD, GLUTs may represent a promising therapeutic target.

In addition to common metabolic diseases such as T2D and NAFLD, as well as cancer, abnormalities in GLUT function may also lead to certain rare genetic disorders, which are often associated with congenital metabolic dysfunction. Although epilepsy is typically considered a neurological disorder, certain types of epilepsy are closely related to metabolic abnormalities, particularly in cases of inherited metabolic diseases. For example, mitochondrial dysfunction, amino acid metabolism disorders (such as phenylketonuria), and GLUT1 deficiency syndrome (GLUT1 DS) can also trigger epileptic seizures. A congenital defect in *SLC2A1* has been shown to cause epilepsy, along with developmental delays, microcephaly, dyskinesia, and other neurological symptoms (Haridas and Kossoff, 2022). Regarding other rare metabolic diseases, *SLC2A2* mutations can lead to the rare genetic disorder Fanconi-Bickel syndrome (Setoodeh and Rabbani, 2012; Shah et al., 2016), which causes excessive excretion of glucose, bicarbonate, phosphate, uric acid, potassium, and certain amino acids in the urine.

#### 3.1.2 SLC5 and dysregulation of glucose transport

Sodium-glucose cotransporters belong to the *SLC5* gene family and mainly mediate glucose transport. Unlike GLUTs, SGLTs are secondary active transport proteins that rely on the sodium ion concentration gradient to actively transport glucose. They co-transport glucose with sodium ions from a region of lower concentration to a region of higher concentration, a process that requires energy expenditure (Han et al., 2022). To date, six subtypes of SGLT proteins, namely, SGLT1-6, which are encoded by the *SLC5A1*, *SLC5A2*, *SLC5A4*, *SLC5A9*, *SLC5A10* and *SLC5A11* genes, respectively, have been identified. Among these proteins, SGLT1 (SLC5A1) and SGLT2 (SLC5A2) are the most studied. SGLT1 is expressed in the brush border membrane of enterocytes and on the apical membranes of kidney of epithelial cells of the proximal straight tubules. SGLT2 is mostly expressed on the apical membranes of the early segment of the proximal tubule in the kidney (Ghezzi et al., 2018).

In preclinical studies, both SGLT1 and SGLT2 have been identified as potential targets for the treatment of glucose metabolism abnormalities due to their glucose transport functions. Osswald et al. reported that increased SGLT1 expression in the intestinal epithelium of mice regulated by RSC1A1 (Regulatory Solute Carrier Protein 1A1) enhanced glucose absorption and contributed to non-leptin-mediated obesity (Osswald et al., 2005). Similarly, *Slc5a2* knockout mice exhibited both significant glycosuria and improved glycemic control (Powell et al., 2013). However, the regulation of glucose absorption by SGLT1 in the gastrointestinal and renal tracts may result in additional gastrointestinal side effects of SGLT1 inhibition due to the influence on normal glucose absorption.

In addition to the widely studied SGLT1 and SGLT2, SGLT5 has also been linked to abnormal glucose metabolism, including diabetes and obesity. The SGLT5 protein is located in the apical membrane of kidney tubule epithelium and primarily transports mannose and fructose, with glucose and galactose being transported to a lesser extent. Under a high-fat diet, *Slc5a10* knockout mice exhibited more severe hepatic steatosis compared to wild-type mice, indicating a previously unrecognized link between renal fructose reabsorption and hepatic lipid metabolism mediated by SGLT5 (Fukuzawa et al., 2013). A genome-wide association study of 1,5-anhydroglucitol, a biomarker of hyperglycemic fluctuations linked to diabetic complications, identified SLC5A10 as a novel locus associated with glucose metabolism (Li et al., 2017).

### 3.2 Bile acid transport and metabolic dysregulation

Bile acids (BAs) are initially synthesized from cholesterol in the liver as primary BAs and excreted into the bile. Primary BAs are then metabolized by gut microbiota to form secondary BAs. Both primary and secondary BAs can be conjugated with glycine or taurine are classified as conjugated bile acids. While unconjugated bile acids can diffuse across cell membranes, conjugated bile acids require active transport. The bile salt export pump (BSEP, also known as ABCB11), located on the canalicular membrane of hepatocytes, transfers conjugated bile acids from hepatocytes into bile canaliculi. The Na⁺/taurocholate cotransporting polypeptide (NTCP, encoded by the *SLC10A1* gene), located on the basolateral membrane of hepatocytes, actively transports conjugated and some unconjugated bile acids from portal venous blood into hepatocytes. Similarly, the apical sodium-dependent bile acid transporter (ASBT, encoded by the *SLC10A2* gene), located on the brush-border membrane of intestinal epithelial cells in the terminal ileum, actively absorbs conjugated and unconjugated bile acids from the intestinal lumen into enterocytes. This process contributes to the enterohepatic circulation of bile acids (Zhou et al., 2014). As a metabolite and a signaling molecule, bile acids can activate various receptors and signaling pathways in the liver and other organ tissues, and play a role in regulating blood glucose and lipid metabolism, increasing insulin sensitivity, and maintaining energy homeostasis (Huang et al., 2019). Albaugh et al. (2017) mentioned that bile acids involved in regulating glucose and lipid metabolism through the activation of the farnesol X receptor (FXR) and the G protein-coupled bile acid receptor (GBPAR, TGR5). The disorder of bile acid metabolism *in vivo* can lead to the occurrence of metabolic diseases such as obesity, T2D, NAFLD, cholestasis, and gallstones (Cai et al., 2022).

#### 3.2.1 SLC10 and dysregulation of bile acid transport

The SLC10 protein family consists of seven members, including NTCP, ASBT, the sodium ion-dependent organic anion transporter (SOAT, encoded by the *SLC10A6* gene), SLC10A3, SLC10A4, SLC10A5, and SLC10A7 (Claro da Silva et al., 2013). Among them, NTCP and ASBT are mainly responsible for the transport of bile acids. NTCP is an influx transporter located exclusively on the basolateral membrane of hepatocytes. ASBT is located on the apical membrane of ileal enterocytes, renal proximal tubule cells, bile duct cells and gallbladder epithelial cells, which are responsible for absorbing bile acids from the intestine into intestinal cells. Growing evidence indicates that bile acids play a critical role in metabolic diseases (Thomas et al., 2008; McGlone and Bloom, 2019). Bile acids can improve insulin sensitivity and reduce fat accumulation, thereby decreasing the occurrence of obesity, T2D, and NAFLD.

NTCP (SLC10A1) and ASBT (SLC10A2), as bile acid transporters, are indeed associated with the occurrence and development of metabolic diseases and may be potential therapeutic targets (Donkers et al., 2019b; Yang et al., 2020). Mutations in the human *SLC10A2* gene may lead to bile acid malabsorption, enterohepatic circulation disruption, and lower plasma cholesterol (Slijepcevic and van de Graaf, 2017), and ASBT deficiency may lead to inflammatory bowel disease, constipation and alagille syndrome, familial hypertriglyceridemia, congenital chronic diarrhea, irritable bowel syndrome (IBS), NASH (Dawson, 2011). Studies have shown that in animal models of diabetes, ASBT inhibitors can effectively improve insulin sensitivity, reduce blood glucose and increase insulin levels (Chen et al., 2012; Wu et al., 2013). In 2015, Vaz et al. reported the first case of *SLC10A1* deficiency, which presented with hypercholesterolemia and normal levels of bilirubin, dysplasia but no pruritus or jaundice (Vaz et al., 2015). However, unlike patients with ASBT deficiency, most patients with NTCP deficiency turned out to be asymptomatic.

Mutations in other proteins within the SLC10 family can result in inherited metabolic diseases. It has been reported that *SLC10A7* mutation can lead to the occurrence of congenital glycosylation disorder, a rare hereditary metabolic disorder (Durin et al., 2022). Related patients present with skeletal dysplasia, accompanied by multiple large joint dislocations, short stature and enamel imperfection. This effect may be mediated by glycosaminoglycan deficiency.

In addition to their role in contributing to metabolic diseases, bile acids are essential for the absorption and metabolism of fatty acids. As a result, the SLC10 family is linked to lipid metabolism disorders. Donkers et al. reported that *Slc10a1* deficiency could prevent obesity and hepatic steatosis induced by a high-fat diet in mice, which may result from the decrease of bile acid uptake (Donkers et al., 2019a). The increase of peripheral bile acid, which activates FXR, induces the expression of *Tgr5* gene, stimulates the secretion of glucagon peptide-1, and thus improves liver glucose and lipid metabolism (Chiang and Ferrell, 2020). Therefore, targeting NTCP-mediated bile acid uptake may offer a novel approach to treating obesity and obesity-related cirrhosis. Several studies have shown that the application of ASBT inhibitors can ameliorate high-fat diet-induced hepatic steatosis, reduce hepatic lipid accumulation, and improve insulin sensitivity in NAFLD mice (Rao et al., 2016; Ge et al., 2019). This effect was verified in *Slc10a2* knockout mice (Kunst et al., 2022). Oral administration of an ASBT inhibitor protected hamsters from high-fat diet-induced NAFLD by regulating bile acids and lipid homeostasis (Ge et al., 2019). Inhibition of ASBT also changes the composition of bile acids in the liver, resulting in the increase of bile acids with FXR activation. When FXR is activated in the liver, bile acid synthesis increases and the expression of lipid synthesis gene *Srebp1* is reduced, thereby reducing cholesterol accumulation (Rao et al., 2016).

### 3.3 Uric acid transport and metabolic dysregulation

Uric acid, derived from the metabolism of purines, is excreted through the kidneys, helping to maintain a dynamic balance of uric acid levels within the body. However, excessive intake of purine-rich foods within a short period can overwhelm the body’s ability to metabolize and eliminate uric acid. Additionally, genetic defects or kidney dysfunction can lead to uric acid accumulation in the blood, resulting in hyperuricemia and gout. SLC2A9 (GLUT9) and SLC16A9 (MCT9) are directly identified as urate transporters, while certain members of the SLC17 and SLC22 protein families are also considered primary transporters of urate. Therefore, these transporters are closely associated with the development of hyperuricemia and gout.

#### 3.3.1 SLC17 and dysregulation of urate transport

The SLC17 family can be classified based on substrate transport into the following types: Na^+^-dependent phosphate transporters (NPT1/3-5; SLC17A1–4, primarily responsible for transporting organic anions), a lysosomal acidic sugar transporter (SLC17A5), vesicular glutamate transporters (VGLUT1–3; SLC17A7, SLC17A6, and SLC17A8, respectively), and a vesicular nucleotide transporter (VNUT; SLC17A9) (Reimer, 2013). Among them, SLC17A1 (NPT1) and SLC17A3 (NPT4) are considered the primary transporters of urate (Andrade Sierra and Flores Fonseca, 2018). NPT1, mainly expressed in the kidney, is localized to the apical membrane of the renal proximal tubule. NPT1 mediates both the absorption and excretion of urate. When the cell membrane is depolarized by a high concentration of exogenous potassium (K⁺), NPT1 facilitates the absorption of urate into the cell (Bhatnagar et al., 2016). Conversely, when the cell membrane exhibits a negative potential, NPT1 promotes the efflux of urate (Chiba et al., 2015).

Vávra et al. (2024) discovered the *p.W75C* variant in the *SLC17A1* gene in a cohort of 150 hyperuricemia and gout patients, as well as 150 healthy controls. Functional *in vitro* assays revealed that, unlike the wild-type protein, the *p.W75C* variant significantly limited urate transport activity. Another study in Japan shows that *SLC17A1 rs1165196* variants and the *I269T* mutation significantly reduce the risk of gout due to renal under-excretion and enhance renal urate secretion in patients with gout (Chiba et al., 2015). Based on previous studies, it is clear that directly targeting the SLC17A1 transporter to enhance its transport function or protein levels can increase renal urate excretion and improve the condition of gout patients.

SLC17A3 (NPT4), expressed in the kidneys, liver and small intestine, is mainly located at the apical side of renal tubules, and functions as an apical voltage-driven urate efflux transporter (Jutabha et al., 2010). NPT4 works synergistically with basolateral organic anion transporters 1/3 (OAT1/OAT3) in urate excretion (Jutabha et al., 2011). Serum uric acid is taken up by OAT1/OAT3 into tubular cells, and then intracellular urate is excreted by NPT4 into the urinary lumen (Jutabha et al., 2011). In several cohort studies, the *rs12664474* (Hollis-Moffatt et al., 2012) and *rs1165205* (Dehghan et al., 2008) polymorphisms of the *SLC17A3* gene have been associated with gout, although the results vary across different population cohorts (Wan et al., 2015). Similarly, the *rs9358890* polymorphism of the *SLC17A4* (*NPT5*) gene has also been associated with gout (Togawa et al., 2012).

#### 3.3.2 SLC22 and dysregulation of urate transport

SLC22 transporters fall into at least six subfamilies: OAT (organic anion transporter), OAT-like, OAT-related, OCT (organic cation transporter), OCTN (organic cation/carnitine transporter), and OCT/OCTN-related (Nigam, 2018). Among them, SLC22A6/7/8/11 (OAT1-4) and SLC22A12 (URAT1) are considered to be involved in regulating serum urate levels. SLC22A6 (OAT1) and SLC22A8 (OAT3), as urate/dicarboxylate exchangers, are located on the basolateral side of the proximal tubule for urate uptake and overall function in renal urate secretion (So and Thorens, 2010). SLC22A11 (OAT4) and SLC22A12 (URAT1) are identified as apical transporters in proximal tubule cells (Hagos et al., 2007).In contrast to the other SLC22 transporters, SLC22A7 (OAT2) has a wide tissue distribution. In the kidney, OAT2 is located on the basolateral side of the proximal tubule, serving as a urate uptake transporter (Sakurai, 2013).

A cohort study demonstrated that the *rs45566039* polymorphism of the *SLC22A6* gene reduces urate excretion in patients (Vávra et al., 2022). Deletion of *Slc22a6* and *Slc22a8* in mice leads to decreased urate excretion (Chung and Kim, 2021). *Rs2078267*, *rs2186571*, *rs17299124* and *rs17300741* of *SLC22A11* gene are associated with the renal underexcretion type of gout (Sun et al., 2021). Punicalagin gavage in hyperuricemia mice can downregulate the expression of urate reabsorption proteins URAT1 and GLUT9, while up-regulating the expression of urate excretion protein OAT1, thereby lowering serum uric acid levels (Han et al., 2024). Dysfunctional variants of *SLC22A12* have been identified as pathophysiological causes of renal hypouricaemia. Specifically, the *rs121907892* and *p.W258X* in the SLC22A12 gene significantly reduces the risk of gout (Toyoda et al., 2021). In contrast, the *rs475688* variant (C/C genotype) and the *p.N82N* synonymous mutation in SLC22A12 are positively associated with an increased risk of gout (Pavelcova et al., 2020). Interestingly, administration of the URAT1 inhibitor dotinurad in mice improves hepatic steatosis and insulin resistance induced by a high-fat diet, highlighting the potential role of URAT1 in regulating glucose metabolism (Tanaka et al., 2022).

#### 3.3.3 Other urate transports and metabolic dysregulation

GLUT9, encoded by *SLC2A9*, is primarily expressed in renal tubules responsible for uric acid reabsorption and also facilitates the transport of small amounts of monosaccharides and L-lactate. The decline in its function reduces the kidneys’ ability to excrete uric acid, leading to hyperuricemia. Lin et al. (2024) conducted a population study and found that *SLC2A9 rs3733591-TC + CC* genotypes were closely related to the development of gout. Through organoid-based assessments, Wu et al. (2023) demonstrated that *SLC2A9 rs16890979* reduces uric acid absorption. Other genetic association studys also revealed that certain single nucleotide polymorphisms (SNPs) in the *SLC2A9* gene are associated with uric acid levels (Jeroncić et al., 2010; Polasek et al., 2010; Lukkunaprasit et al., 2020).

SLC16A9 (MCT9) is primarily localized in the small intestine and kidneys, and mainly transports small organic acids such as lactate, creatine, and pyruvate. Although the relationship between SLC16A9 and urate is not yet clear, several cohort studies have found that variants of *SLC16A9 rs2242206* (Nakayama et al., 2013) and *rs1171614* (Butler et al., 2021) significantly increase the risk of renal overload gout.

### 3.4 Lipid transport and metabolic dysregulation

Fatty acids are transported into the epithelial cells of the small intestine via fatty acid transport proteins (FATPs, encoded by *SLC27*). Excess body fat accumulation can lead to overweight and obesity, which increases the risk of NAFLD, T2D, insulin resistance, hypertension, hyperlipidemia, and cardiovascular disease. Given the modern human diet, which is often high in salt, carbohydrate, and fat, FATPs, primarily responsible for the absorption and transport of fatty acids into cells, are major factors in the rising incidence of lipid metabolism disorders.

#### 3.4.1 SLC27 and dysregulation of lipid transport

SLC27, also known as the family of fatty acid transporter proteins, was originally discovered in the mouse 3T3-L1 adipocyte cDNA library using cloning technology (Schaffer and Lodish, 1994). Currently, there are six members of the family (*SLC27A1–6*) that encode the FATP1–6 proteins. Although FATPs share sequence similarity, their expression in the human body is tissue specific. For instance, FATP1 and FATP3 are distributed across nearly all organs. FATP4 is predominantly expressed in the colon and small intestine, while FATP6 expression is higher in the adrenal glands, heart, and testes. In contrast, FATP2 is mostly found in the liver and kidneys, and FATP5 is almost exclusively found in the liver.

As a major transporter of fatty acids, FATPs have been shown to be strongly associated with the development of NAFLD. Liver-specific knockdown of *Fatp2* using shRNA improved high-fat diet-induced hepatic steatosis and increased insulin sensitivity in mice (Falcon et al., 2010). Mice with the hepatic *Slc27a5* gene silenced showed that inhibiting the expression of FATP5 reversed NAFLD induced by a high-fat diet and improved hyperglycemia (Doege et al., 2008). In addition, a population study suggested that polymorphisms in the *SLC27A5* promoter may be associated with the clinical symptoms of hepatic steatosis and metabolic syndrome (Auinger et al., 2010).As the research progressed, people realized that excessive accumulation of fatty acids can cause insulin resistance. Emerging evidence indicates that fatty acid transporters contribute to the development of T2D, with skeletal muscle insulin resistance being a critical factor in its pathogenesis. Kim et al. reported that knockout of *Slc27a1* gene inhibited diet-induced insulin resistance in skeletal muscle of mice (Kim et al., 2004). Khan et al. reported that knocking down *Slc27a2* reduced the abnormally elevated blood glucose concentration in model mice (Khan et al., 2020), indicating that FATP2 could regulate blood glucose homeostasis and the progression of diabetic nephropathy. Thus, FATPs may be a new potential therapeutic target for treating diabetes.

### 3.5 Monocarboxylate transport and metabolic dysregulation

#### 3.5.1 SLC16 and dysregulation of monocarboxylate transport

Monocarboxylate is a major participant in cellular energy metabolism and a product of anabolic substrates and catabolic pathways in various tissues. The entry and exit of monocarboxylic acid through cells are mainly mediated by monocarboxylate transporter protein (MCT). This protein family is encoded by the *SLC16* family of genes, which include 14 members that regulate the cellular acid‒base balance and participate in various metabolic pathways (Felmlee et al., 2020), including glucose homeostasis and gluconeogenesis.

SLC16A1 (MCT1), the first and most common member of the SLC16 family, is widely distributed in various tissues and cells (Leu et al., 2021). MCT1 is expressed in both the apical and basolateral membrane of cells (Sivaprakasam et al., 2017), and is involved in the transmembrane transport of lactate, pyruvate, and ketone bodies. Studies have shown that MCT1 is related to human metabolic characteristics and involved in regulating insulin secretion (Galcheva et al., 2018). Constructing *Slc16a1* knockout mouse model demonstrated that SLC16A1 may contribute to the treatment of exercise-induced hyperinsulinemia (Pullen et al., 2012), at the same time, it may exacerbate ketoacidosis (Balasubramaniam et al., 2016).

MCT10, encoded by the *SLC16A10* gene, transports aromatic amino acids and is highly expressed in skeletal muscle and heart. A genomic study of branched-chain amino acid metabolism in patients at different stages of NAFLD revealed that *SLC16A10* gene expression was reduced in NASH (Lake et al., 2015); however, it is necessary to expand the sample size and include multiple populations for genetic studies and related regulatory mechanisms. Another member of the SLC16 family, SLC16A3/MCT4, has been found to be associated with diabetic nephropathy (Lokman et al., 2011).

Recent studies have revealed that mutations in the *SLC16A11* gene encoding MCT11 are associated with an increased risk of developing T2D. T2D susceptibility mutations in the SLC16A11 coding region produce gain-of-function mutant proteins, resulting in the upregulation of hepatic Lipin1 protein expression and an abnormal accumulation of lipid droplets, ultimately contributing to the onset of T2D (Zhao et al., 2019b). Otherwise, the knockdown of *Slc16a11* improved glucose tolerance and insulin sensitivity in mice fed a high-fat diet (Zhang et al., 2021). Although the specific mechanisms by which SLC16A11 plays a role in T2D risk and progression have not yet been fully elucidated, the evidence of its clinical impact on T2D warrants further experimental investigation while also potentially providing new ideas for other metabolic diseases. As a member of the same family, the *SLC16A13* gene encoding MCT13 has also recently been identified as a potential susceptibility gene for diabetes. The *rs312457* genotype of the *SLC16A13* gene was associated with the occurrence of diabetes in a Chinese population (Zheng et al., 2021).The knockdown of *Slc16a13* ameliorated hepatic lipid accumulation and insulin resistance in mice (Schumann et al., 2021), indicating that SLC16A13 may be a potential therapeutic target for both T2D and NAFLD.

In addition to the SLC2 family, the SLC16 family is also closely associated with tumorigenesis. Cancer cells undergoing aerobic glycolysis produce lactate and release it into the extracellular compartment (Walenta et al., 2001), altering the tumor microenvironment. Accumulated lactate not only induces metabolic reprogramming but also promotes tumor inflammation and acts as a signaling molecule to stimulate tumor angiogenesis (Chen et al., 2024). Additionally, it affects the function, differentiation, and metabolism of immune cells, contributing to tumor immune evasion and promoting tumor progression (Doherty and Cleveland, 2013; Wang et al., 2021). The MCT family, which is primarily responsible for the transmembrane transport of monocarboxylates (such as lactate and pyruvate), is involved in metabolic reprogramming, adaptation to acidic environments, and intercellular communication in cancer cells.

Of all the MCT proteins, MCT1 and MCT4 have been the most studied in relation to tumors. Xiaowei She et al. found that MCT1 methylation modification was upregulated and positively correlated with tumor progression and overall survival in colorectal cancer (She et al., 2023). Marte Eilertsen et al. put forward the view that MCT1 is a candidate marker for prognostic stratification in non-small cell lung cancer (Eilertsen et al., 2014). Other researchers found that silencing *SLC16A1* inhibited breast cancer (Weng et al., 2019), bladder cancer (Zhang et al., 2018) and lung cancer (Liu et al., 2023) progression. Meanwhile, MCT4 is found to be upregulated in triple-negative breast cancer (Duan et al., 2022), bladder cancer (Zhong et al., 2023) and prostate cancer (Sun et al., 2019). Xiao Hu et al. found that the mutation of Lys448 (K448) inhibited the SUMOylation of MCT4, promoting MCT4 degradation, thereby slowing the progression of breast cancer (Hu et al., 2021). Although there are few studies on other members of the SLC16 family and tumors, some reports have described the upregulated expression of MCT family proteins in tumor tissues. For example, MCT5 expression was upregulated in colon cancer (Lin et al., 2019). MCT3, MCT8, MCT9 are upregulated in breast cancer (Sohrabi et al., 2021). MCT8 was downregulated in thyroid cancers (Badziong et al., 2017). These findings demonstrate the potential of SLC16 family proteins as tumor markers and therapeutic targets. Targeting MCT-mediated lactic acid influx and efflux in cancer cells has become an effective strategy for inhibiting tumor cell growth *in vitro* (Payen et al., 2020). MCT1 inhibitors are in advanced stages of drug development (Phase I/II clinical trials), while MCT4 inhibitors remain in the drug discovery phase (Singh et al., 2023). Combining dual MCT1 and MCT4 inhibitors with metformin depletes NAD^+^ in cancer cells, resulting in synthetic lethality (Benjamin et al., 2018). Inhibition of MCT1 or MCT4 in combination with chemotherapy has demonstrated additive or synergistic effects (Zhao et al., 2014; Lee et al., 2016b). Co-administration of the MCT4 inhibitor and immune checkpoint blockade increased intratumoral pH, improved leukocyte infiltration and T-cell activation, delayed tumor growth, and prolonged survival *in vivo* (Babl et al., 2023).

### 3.6 Tricarboxylic acid cycle intermediates transport dysregulation

Citrate, succinate, and α-ketoglutarate, intermediates of the tricarboxylic acid cycle (TCA), are key regulators of energy metabolism, transported by sodium-dependent transporters sodium-dependent dicarboxylic acid transporters, including SLC13A2, SLC13A3, and SLC13A5. The metabolic disorders of these intermediates can lead to conditions such as intestinal inflammation (Connors et al., 2018), cancer, insulin secretion, histone acetylation, neurological diseases, and NAFLD (Iacobazzi and Infantino, 2014).

Pyruvate metabolism mainly occurs in the mitochondrial TCA and is closely related to cellular energy metabolism. At the same time, pyruvate is also related to carbohydrate and fat metabolism (Deja et al., 2024). Pyruvate enters mitochondria and is converted into acetoacetate, amino acids, and other substances under the catalytic action of various enzymes. The former is not only a key intermediate of gluconeogenesis, but also can synthesize citric acid with acetyl CoA as a raw material for the synthesis of fatty acid and cholesterol. The disorders of lactate and pyruvate metabolism can lead to acidosis, hyperketonemia, psychomotor disorders, etc. (Gray et al., 2014).

Inorganic anions (sulfate, sulfite, thiosulfate, and phosphate), dicarboxylates (*e. g.*, malonate, malate, and citrate), aspartate, glutamate, and tricarboxylate are all transport substrates for the uncoupling protein (UCP) encoded by the mitochondrial carrier protein family *SLC25*. These substances are involved in almost important biological metabolic processes, such as oxidative phosphorylation, citric acid cycle, fatty acid oxidation, gluconeogenesis, lipogenesis, urea synthesis, amino acid metabolism, and thermogenesis. Glycolysis/gluconeogenesis and tricarboxylic acid circulating metabolites are associated with T2D (Guasch-Ferré et al., 2020), and citrate is reported (Yadikar et al., 2022) to modulate insulin sensitivity for further treatment of hyperlipidemia-induced glucose metabolism disorders. Glycolysis/gluconeogenesis and tricarboxylic acid cycle metabolites are also involved in the hepatic lipid production process and are associated with NAFLD progression (Zhao et al., 2020).

#### 3.6.1 SLC13 and dysregulation of intermediates of the tricarboxylic acid cycle transport

As mentioned earlier, sodium-dependent dicarboxylate transporters, including SLC13A2, SLC13A3, and SLC13A5, which mainly transport intermediates of the tricarboxylic acid cycle, such as citrate, succinate and α-ketoglutarate.

The SLC13 family consists of five members, SLC13A1–5 and comprises transporters (Bergeron et al., 2013) with 8–13 transmembrane domains in their structures. According to their functions, members of this family can be divided into two main types (Markovich, 2012): (1) sodium sulfate cotransporters (NAS), which include both SLC13A1 and SLC13A4, the main transporters of sulfate, selenate and thiosulfate; and (2) sodium-dependent dicarboxylate transporters (NADC), including SLC13A2, SLC13A3, and SLC13A5, which mainly transport intermediates of the tricarboxylic acid cycle, such as citrate, succinate, and α-ketoglutarate.

The NADC1 protein encoded by the *SLC13A2* gene is mainly found in the apical membranes (Pajor, 2014) of the proximal renal tubules cells and small intestinal cells, and the main physiological role of NADC1 protein is responsible for citric acid reabsorption and regulation of citric acid excretion in the proximal renal tubules (Okamoto et al., 2007; Chi et al., 2024). Current research shows that NADC1 is mainly related to the formation of kidney stones (Yang et al., 2021). Patients with kidney stones often have low levels of urine citrate. Blocking NADC1 can inhibit the renal reuptake of citric acid and increase the renal excretion of citric acid, preventing calcium deposition from forming kidney stones. In addition, there is no evidence that SLC13A2 polymorphisms are directly related to obesity, diabetes, insulin resistance, or fatty liver in humans.

SLC13A3, also known as NADC3 or SDCT2 (high-affinity sodium-dependent dicarboxylate co-transporter), is present in various tissues but is mainly located on the basolateral membrane in the kidney and liver (Pajor et al., 2001; Schlessinger et al., 2014). Genome-wide association studies revealed that NADC3 may be involved in diabetic nephropathy as well as chronic kidney disease (Bento et al., 2008; Ju et al., 2009; Nanayakkara et al., 2014). At present, there are very few reports on the role of NADC3 in metabolic diseases such as obesity, diabetes and NAFLD. Only one article describing the transcriptomic analysis of the livers of diabetic rats found that the expression level of the *Slc13a3* gene in the livers of diabetic rats increased, and its expression level recovered after intervention treatment (Li et al., 2020), suggesting that it may be highly correlated with glucose metabolism. A connection between NADC3 and NAFLD has not yet been reported.

The protein NACT, encoded by the *SLC13A5* gene, is a sodium-dependent citric acid transporter and a switch that controls citric acid transport (Akhtar et al., 2023). NACT is found on the basolateral membrane of hepatocytes and on the plasma membrane of neurons and astrocytes. The association between NACT and metabolic disease has been well validated in animal models. The researchers verified that siRNA silencing of *Slc13a5* or knockout of *Slc13a5* in the liver of mice can improve obesity, insulin resistance, and liver steatosis induced by a high-fat diet. This may be related to the fact that decreased NACT expression promotes liver mitochondrial biosynthesis, enhances lipid oxidation, and reduces *de novo* lipogenesis in the liver (Birkenfeld et al., 2011; Brachs et al., 2016). NACT inhibitors block NACT-mediated citrate uptake in mice and humans, thereby reducing liver steatosis and body fat, and improving blood glucose regulation (Zahn et al., 2022). This result is consistent with that obtained by knocking out *Slc13a5* gene in mice. More importantly, Loeffelholz et al. reported that *SLC13A5* gene expression levels were significantly increased and independently correlated with hepatic steatosis in liver samples from NAFLD patients with obesity for the first time (von Loeffelholz et al., 2017), revealing the relationship between NACT and hepatic steatosis in humans.

#### 3.6.2 SLC25 and dysregulation of intermediates of the tricarboxylic acid cycle transport

SLC25 is the largest family of the SLC transporter supergroup and currently consists of 53 members (Rochette et al., 2020). However, there are seven non-function pseudogenes, namely, SLC25A5P1, SLC25A6P1, SLC25A15P, SLC25A20P, and SLC25A51P1-3 (Palmieri, 2013). Most members of this family are localized to the inner mitochondrial membrane and function through the transport of various solutes across the mitochondrial membrane. The nature, size and mode of transport of the transport substrate of SLC25 vary greatly. Studies have shown that SLC25 family proteins participate in many important metabolic pathways, including oxidative phosphorylation, the citric acid cycle, fatty acid oxidation, gluconeogenesis, lipogenesis, urea synthesis, amino acid metabolism and thermogenesis (Palmieri, 2013; Palmieri and Monné, 2016; Zhao et al., 2019a). Therefore, loss or abnormality of SLC25 family proteins can lead to a variety of metabolic diseases.

Uncoupling protein, a well-studied subfamily of the SLC25 family, has been shown to directly transport protons and subsequently regulate energy metabolism. At present, five UCP homologs have been identified in mammals, namely, UCP1 (SLC25A7), UCP2 (SLC25A8), UCP3 (SLC25A9), UCP4 (SLC25A27), and UCP5 (SLC25A14) (Zhao et al., 2019a), among which UCP1–3 are the most well studied. UCP1, also known as thermogenin, is encoded by the *SLC25A7* gene and is abundantly expressed (Schumann et al., 2020) in brown adipose tissue. Studies have shown that brown adipose tissue helps slow the progression of obesity and metabolic syndrome (Chouchani et al., 2016). Loss of brown adipose tissue can lead to obesity, and its activation can reduce fat accumulation. UCP1 is a key protein in brown adipose tissue thermogenesis, so activation of UCP1 can effectively improve obesity and diabetes (Kozak and Anunciado-Koza, 2008; Feldmann et al., 2009; Ikeda et al., 2017). UCP1 can also be used to combat NAFLD and succinate receptor 1-dependent hepatic inflammation (Mills et al., 2021). Although studies have suggested that UCP2 and UCP3 polymorphisms may be associated with obesity, diabetes, or related phenotypes (D'Adamo et al., 2004; Jia et al., 2009; Dalgaard, 2011), their effects on these phenotypes appear to be modest, and the heterogeneity between studies is large (Halsall et al., 2001). To date, the effects of UCP2 and UCP3 on metabolism-related regulation remain elusive and require further evaluation, whereas the function of UCP1 and its connection to metabolic diseases has been well established.

SLC25A1 acts as a specific transporter of citrate. Tan et al. (2020) reported that SLC25A1-specific inhibitor CTPI-2 can reduce macrophage infiltration, prevent steatohepatitis, ameliorate symptoms of inflammatory steatohepatitis, and ameliorate obesity associated with a high-fat diet. Inhibition of SLC25A1 reduces peroxisome proliferator-activated receptor γ (PPARγ) signaling, thereby further reducing gluconeogenic gene expression, normalizing hyperglycemia and glucose intolerance, and leading to inhibition of lipid anabolic processes. Additionally, another study demonstrated that defects in SLC25A1 result in combined D,L-2-hydroxyglutaric aciduria (Nota et al., 2013).

The *SLC25A13* gene encodes a mitochondrial calcium-binding SLC protein in hepatocytes called citrin, also known as AGC2 or CTLN2, which is mainly expressed in the liver and can promote the Ca^2+^-dependent exchange of cytoplasmic glutamate with mitochondrial aspartate. Citrin has three functions: (1) it transports aspartate to the cytosol to participate in the synthesis of protein, nucleic acid and urea; (2) it transports aspartate to the cytosol to transport the NADH generated by glycolysis in the cytosol to the mitochondria and then participates in the metabolism of energy, amino acids, carbohydrates and lipids; and (3) it promotes lactose gluconeogenesis in the formation and utilization of NADH. Studies have shown that patients who develop citrin deficiency or adult-onset type II citrullinemia due to *SLC25A13* gene mutation are prone to steatosis, NASH and even hepatocellular carcinoma (Takagi et al., 2006; Tsai et al., 2006; Komatsu et al., 2008); therefore, the role of SLC25A13 in metabolic diseases may be worthy of further study.

SLC25A20 is also known as carnitine/acylcarnitine translocase (CACT), which can transport acylcarnitine to the mitochondrial matrix to participate in β-oxidation (Indiveri et al., 2011). Clinically, patients with *SLC25A20* deficiency are prone to severe metabolic disorders, which are mainly attributed to disorders in fatty acid oxidation metabolism. However, whether metabolic diseases can be improved by regulating CACT needs further research.

The APC1 protein encoded by the *SLC25A24* gene is widely distributed *in vivo* and promotes the exchange of adenine nucleotides, including ATP-Mg, ATP, ADP, and AMP, as well as the mitochondrial matrix and intercytoplasmic phosphates. This transporter can regulate the adenine nucleotide concentration in the mitochondrial matrix, which in turn affects the mitochondrial adenine nucleotide-dependent enzyme that regulates gluconeogenesis, urea synthesis, mitochondrial DNA replication, transcription, and protein synthesis (Palmieri and Monné, 2016). *Slc25a24* knockout mice are resistant to high-fat diet-induced obesity, as indicated by reduced liver weight and triglyceride deposition in the liver, and a prospective observational study of the population revealed that *SLC25A24* is also a new candidate gene for susceptibility to obesity in humans (Urano et al., 2015). Thus, inhibiting APC1 may improve obesity and NAFLD, but further studies are needed for confirmation.

Bresciani et al. (2022) reported that SLC25A47 is a liver-specific transporter required for maintaining mitochondrial homeostasis in hepatocytes. Mice in which *Slc25a47* was specifically knocked out exhibited a wide range of metabolic changes, from gluconeogenesis and altered liver lipid metabolism to reduced glycogen storage and impaired mitochondrial respiration, leading to increased liver inflammation and fibrosis.

In addition, two other SLC25 family members also appear to be associated with metabolic diseases. *Slc25a25* knockout mice exhibit reduced metabolic efficiency, specifically manifested by resistance to diet-induced obesity and impaired exercise performance (Anunciado-Koza et al., 2011). A population study suggested that the SLC25A40 variant may be associated with hypertriglyceridemia, as whole-gene testing confirmed the link between triglyceride levels and rare, highly conserved coding variants of *SLC25A40* (Rosenthal et al., 2013).

### 3.7 Inorganic salt ions transport dysregulation

Metal salt ions, including sodium, potassium, calcium, zinc, and magnesium, as well as inorganic salts like phosphates, sulfates, thiosulfates, and sulfites, are integral to numerous physiological processes. These ions are critical in maintaining acid-base balance by acting as buffers in the blood and tissues, ensuring that the pH levels remain within a narrow, optimal range. Beyond their role in acid-base homeostasis, they are essential for electrolyte balance, with sodium and potassium playing pivotal roles in maintaining the osmotic balance across cell membranes. Furthermore, zinc and magnesium are indispensable for nerve conduction and muscle function, with magnesium assisting in neuromuscular transmission and muscle contraction, and zinc supporting synaptic function and neurotransmitter release.

#### 3.7.1 SLC13 and dysregulation of inorganic oxides transport

Some proteins in the SLC13 family are major transporters of inorganic oxides ions. As the fourth most abundant anion in human plasma, insufficient sulfate supply suppresses detoxification, increases susceptibility to exogenous substances, and alters the metabolism and activity of multiple endogenous compounds (*e.g.*, hormones, neurotransmitters, and proteoglycans) (Markovich, 2001). Since the inorganic anion sulfate (SO4^2-^) cannot passively cross the cell membrane, all cells rely on plasma membrane sulfate carriers or transport proteins to mediate sulfate influx and efflux. Most of the sulfate is absorbed in the gastrointestinal tract into the body circulation, and the renal proximal tubule is the main site of active sulfate reabsorption. In the renal proximal tubular cells, there are at least two different sulfate transport systems: (1) the Na^+^-sulfate cotransporter (NAS1, encoded by *SLC13A1*) localized in the renal brush border membrane (Markovich and Aronson, 2007), and (2) the sulfate anion transporter (SAT1, encoded by *SLC26A1*), a sulfate-anion exchanger localized in the basolateral membrane in renal proximal tubular cells (Regeer and Markovich, 2004).

SLC13A1, also known as the apical membrane Na^+^-sulfate cotransporter (NAS1), which is mainly expressed in the kidney’s proximal tubule and intestine, mediates sulfate absorption in the proximal tubule of the kidney and in the small intestinal epithelium, thus maintaining the homeostatic balance of sulfate (Markovich, 2014). The literature shows that two single-nucleotide variants of *SLC13A1*, *rs28364172* and *rs138275989*, are closely associated with low serum sulfate levels, known as hyposulfatemia (Tise et al., 2016). Dawson et al. reported that the liver gene expression profile of a *Slc13a1* gene knockout mouse model of hyposulfatemia showed alterations in lipid and cholesterol metabolism, which were specifically manifested as increased levels of liver lipids, serum cholesterol, and low-density lipoprotein and decreased liver glycogen content (Dawson et al., 2006), indicating that SLC13A1 may be related to the development of fatty liver. According to rodent model data, activation of SLC13A1 may be beneficial for metabolism and may become a potential therapeutic target. Because SLC13A1 is involved in multiple physiological processes and the regulatory network is very complex, further studies are needed to identify therapeutic targets through the regulation of this transporter.

#### 3.7.2 SLC30 and dysregulation of zinc transport

As a trace element in the human body, zinc is involved in the growth and development of the body and as a catalyst for enzymes involved in the metabolism of lipids, proteins, and carbohydrates. Zinc ion homeostasis is strictly regulated by the transport process. It is mainly engaged by two types of transporter subfamilies: ZIP (Zrt-and Irt-like protein family) encoded by *SLC39* and ZnT (Zinc transporter ZnT family) encoded by *SLC30*. ZnT transports zinc ions from the cytoplasm to outside cells or organelles. In contrast, ZIP is responsible for transporting zinc ions from outside cells or inside organelles to the cytoplasm (Huang and Tepaamorndech, 2013). The two large subfamilies of proteins coordinate with each other to maintain the homeostatic balance of zinc ions in the body. Zinc is involved in the synthesis, storage, and release of insulin, which suggests the critical role of this microelement in the progression of T2D and metabolic syndrome (Miao et al., 2013; Ahn et al., 2014). Huang et al. (2017) found that long-term zinc supplementation induced hypertrophy of visceral adipose tissue.

The SLC30 family consists of 10 members. When the intracellular zinc level is too high, the SLC30 family transports zinc ions out of the cytoplasm or into the organelles, thereby reducing the intracellular zinc level (Palmiter and Huang, 2004). SLC30 family proteins are closely linked to diabetes, Alzheimer’s disease, or Parkinson’s disease (Hara et al., 2017). In terms of metabolic diseases, the most prominent member of the SLC30 family is SLC30A8 (ZnT8), which is located mainly in the insulin-secreting vesicle membrane of β cells in the pancreas and provides the zinc for insulin synthesis (Chimienti et al., 2004). Numerous studies have shown that ZnT8 is closely related to type 1 diabetes mellitus (T1D) and T2D (Daniels et al., 2020; Soltanian et al., 2020; Xu et al., 2021). Abnormalities in ZnT8 can easily lead to islet β-cell dysfunction, but the specific molecular mechanism by which ZnT8 functions as an autoantigen of T1D and a susceptibility gene for T2D requires further investigation, as it eventually leads to diabetes. In addition, it has been found that the overexpression of SLC30A8 in islet α cells leads to the inhibition of glucagon secretion, which may have potential benefits for treating T2D (Solomou et al., 2016).

ZnT7, encoded by the *SLC30A7* gene, is located at the plasma and organelle membranes in most tissues throughout the body. It has been found that male *Slc30a7* knockout mice fed a high-fat diet developed symptoms of metabolic disorders, including insulin resistance, abnormal glucose tolerance, and hyperglycemia (Huang et al., 2012). The ZnT3 protein, encoded by the *SLC30A3* gene, is located mainly in the brain, and to date, the SLC30A3 polymorphism has not been linked to human metabolic diseases but has been linked to schizophrenia and Alzheimer’s disease (Rovelet-Lecrux et al., 2012; Perez-Becerril et al., 2014). However, Smidt et al. studied the role of ZnT3 in metabolism through cell and mouse models and reported that the expression level of *SLC30A3* in islet β cells was higher with increasing glucose concentration. Silencing *Slc30a3* reduced the expression and secretion of insulin in islet β cells (Smidt et al., 2009).

#### 3.7.3 SLC39 and dysregulation of zinc transport

There are 14 members of the SLC39 family, known as the zinc-regulated transporters and iron-regulated transporter-like proteins (ZIPs). Unlike SLC30 family proteins, when the cytoplasmic zinc concentration is insufficient, the SLC39 family transports zinc ions into the cytoplasm, thereby increasing the cytoplasmic zinc concentration (Palmiter and Huang, 2004). Most ZIPs are localized to the plasma membrane and mediate zinc uptake into the cytoplasm. Some specific ZIPs are localized in cellular compartments and mediate the release of zinc from these compartments, such as ZIP4 (encoded by *SLC39A4*) expressed on the apical membrane of intestinal epithelial cells and ZIP5 (encoded by *SLC39A5*) localized to the basolateral membrane of intestinal epithelial cells and acinar cells. In contrast, ZIP7 and ZIP13 are localized to endoplasmic reticulum or Golgi bodies (Nagamatsu et al., 2022).

Previous studies have shown that the pathological features caused by the altered function of the SLC39 transporter mainly include abnormal development of embryos and immune cells (Jeong and Eide, 2013). However, with in-depth research in recent years, it has been found that some members of the SLC39 family may also be involved in metabolic diseases. For example, recent studies have shown that ZIP5 may be involved in regulating the glucose sensing and insulin secretion of islet β cells by silencing the expression of GLUT2, which is mediated by SIRT1 and PGC-1α. A deficiency in *SLC39A5* weakens glucose sensitivity and reduces insulin secretion, indicating that SLC39A5 has potential as a therapeutic target for diabetes-related metabolic diseases (Wang et al., 2019).

Similarly, the role of ZIP13 (*SLC39A13*) in adipocyte browning has received much attention in recent years. ZIP13 has been identified as an important regulator of beige adipocyte differentiation and negatively regulates C/EBP-β protein levels (Fukada et al., 2008). ZIP13 loss-of-function mutations can lead to Ehlers-Danlos syndrome in affected individuals (Bin et al., 2014).

ZIP14 (*SLC39A14*) also appears to be closely associated with metabolic diseases. By constructing an *Slc39a14* knockout mouse model, it was found that a high-fat diet induced remission of insulin resistance. Further glucose homeostasis experiments showed that zinc deficiency impaired gluconeogenesis in the liver cells of *Slc39a14* knockout mice (Aydemir et al., 2016). Troche et al. found that ZIP14 is involved in not only the synthesis of adipokines in adipose tissue but also the development of adipocytes and the transport of zinc ions during fat deposition (Troche et al., 2016). ZIP14 is highly expressed in human pancreatic β cells (Lawson et al., 2018). The increase in ZIP14-mediated uptake of non-transferrin-bound iron into pancreatic β cells may lead to cell damage and trigger diabetes (Coffey and Knutson, 2017). Although current studies shed light on ZIP14’s function, its precise regulation in different metabolic organs requires further investigation. Continued exploration of ZIP14 may offer new insights and therapeutic strategies for metabolic diseases.

**FIGURE 2:**
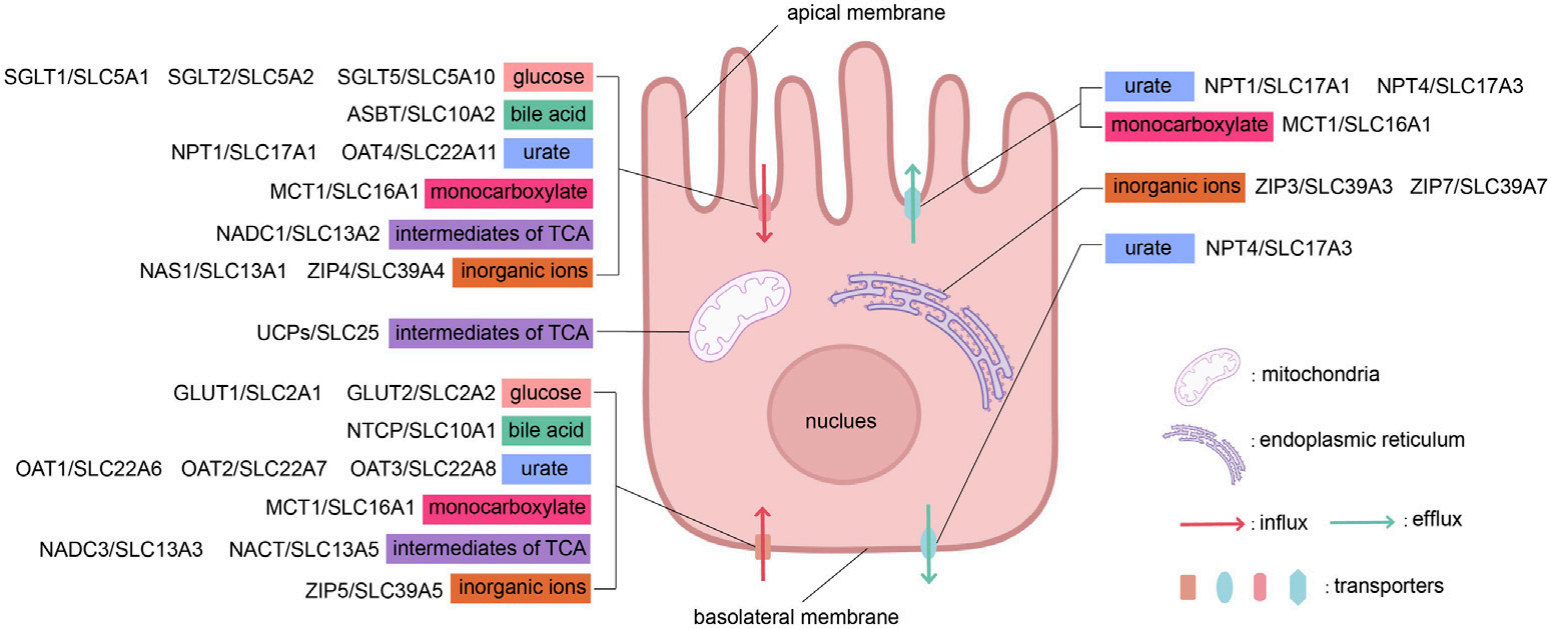
The influx and efflux transport of substances across apical and basolateral membranes by different types of transporters. This figure was created using BioRender.

The subcellular localization of SLC family transporters located in epithelial cells, as mentioned in the text, is shown in Figure 2.

## 4 Current status and prospect of SLC clinical application

### 4.1 SLC inhibitors in clinical applications

Among the more than 450 SLC protein family members discovered thus far, approximately half of the SLC protein gene mutations are associated with disease; however, currently, there are only approximately 20 drugs targeting SLC transporters, such as the SGLT2 inhibitors canagliflozin, dapagliflozin, ertugliflozin, ipragliflozin, tofogliflozin, luseogliflozin and empagliflozin (Tentolouris et al., 2019); the first marketed dual SGLT1 and SGLT2 inhibitor sotagliflozin (Rendell, 2018); henagliflozin, as a potent SGLT2 inhibitor, has been approved for the treatment of T2D in China and is also being studied for the treatment of NAFLD. High-quality evidence suggests that SGLT2 inhibitors may increase the risk of diabetic ketoacidosis in patients with T2D. The SLC9A3 inhibitor tenapanor for the treatment of hyperphosphatemia in patients with constipated IBS and dialysis chronic kidney disease or end-stage renal disease (Markham, 2019). NTCP-inhibitor myrcludex B (also known as Bulevirtide) has been approved in Europe for the treatment of chronic hepatitis D (Kang and Syed, 2020). Lesinurad, an SLC22A12 inhibitor approved by the FDA for the treatment of gout (Deeks, 2017). It was later withdrawn from the market in 2019 due to acute kidney injury. Another SLC22A12 inhibitor dotinurad has been approved in some Asian countries for the treatment of hyperuricemia and gout. Thiazide diuretics, such as chlorothiazide, indamine, and other drugs, act on the renal tubule sodium-chloride cotransporter (SLC12A3), inhibit the reabsorption of Na^+^ and Cl^−^ and play a role in sodium diuresis, and are widely used in the treatment of hypertension. Elobixibat is an ASBT inhibitor developed by Albireo, and is approved in Japan for the treatment of chronic constipation and IBS.

In addition to the approved drugs mentioned above, many drugs targeting SLC family proteins have entered the clinical trial stage. Triheptanoin has entered a phase II trial for the treatment of GLUT1 deficiency syndrome, which is caused by a heterozygous mutation in the *SLC2A1* gene (Striano et al., 2022). It works by providing an alternative energy source to glucose, helping to compensate for impaired glucose transport into the brain and improving neurological function. However, a phase III crossover study on the use of triheptanoin to treat paroxysmal movement disorders in GLUT1 deficiency syndrome showed that triheptanoin had no significant therapeutic effect (De Giorgis et al., 2024). Clinical data analysis from Italy indicates that triheptanoin treatment significantly reduces the frequency of metabolic decompensation episodes requiring hospitalization in patients with long-chain fatty acid oxidation disorders (LC-FAOD) (Porta et al., 2024). Mutations in the SLC25A20 gene lead to CACT deficiency, which is a cause of LC-FAOD. A SLC22A12 inhibitor verinurad are in phase II clinical trials for the treatment of hyperuricemia and chronic kidney disease. The SGLT1 inhibitor mizagliflozin has entered phase II clinical trials for the treatment of chronic constipation (Fukudo et al., 2018). NTCP-inhibitor myrcludex B is a potential drug for phase III clinical trials for treatment of hepatitis B virus (Cheng et al., 2021). Clodronate, an SLC25A4, SLC25A5, and SLC25A6 inhibitor, is commonly used in the treatment of osteoporosis (Lehenkari et al., 2002). Clodronate has recently been identified as an effective inhibitor of vesicle nucleotide transporter (VNUT/SLC17A9), and is a potential drug for the relief of chronic neuropathic pain (Moriyama and Nomura, 2018). Interestingly, glutamine, the transport substrate of VNUT (SLC17A9), plays a regulatory role in insulin secretion, and therefore, clodronate has been demonstrated to have therapeutic effects on T2D and NASH in mice models (Hasuzawa et al., 2021).

### 4.2 Other SLC-related clinical applications

In addition to transporter inhibitors directly targeting SLC proteins for the treatment of metabolic diseases, researchers and clinicians have found that SLC transporters can also be used as probes for disease diagnosis and to indirectly enhance drug selectivity or efficacy through SLC transporters. The use of glucose transporters (such as SLC2A1) in ^18^F-deoxyglucose positron emission tomography is a commonly used imaging technique in medical imaging, suitable for the diagnosis of various cancers (Massardo et al., 2007). Radiolabeled markers such as ^18^F-FDOPA (Rozenblum et al., 2020) and ^18^F-AMT (Krasikova et al., 2020) accumulate inside tumor cells via the l-type amino acid transporter 1 (SLC7A5; LAT1) and are used for brain function imaging and tumor metabolism research. ^11^C-metformin, primarily transported by the basolateral organic cation transporter 1 (SLC22A1; OCT1) in the liver, the basolateral organic cation transporter 2 (SLC22A2; OCT2) and the apical multidrug and toxin extruders (SLC47A1/2; MATE1/2) in the kidneys, is considered a potential PET probe to accurately quantify kidney function (Gormsen et al., 2016). Through the transport of ^18^F-fluoroglutamine via SLC1A5 (ASCT2), PET imaging is used to diagnose various solid tumors (Hassanein et al., 2016). Similarly, SLC15A1/2 (PEPT1/2) mediates the transport of ^11^C-glycylsarcosine for PET imaging to diagnose tumors (Mitsuoka et al., 2008), while SLC10A1 (NTCP) facilitates the transport of ^18^F-fluorocholic acid analogs to evaluate hepatic bile transport function (De Lombaerde et al., 2019). These applications leverage the specificity of SLC family members and their substrates to deliver radioactive tracers to target tissues and organs for imaging. This approach has gradually gained widespread use in clinical oncology diagnosis and organ function assessment.

Tumor cells have an increased demand for amino acids and glucose due to their rapid proliferation rate. Therefore, amino acid transporters, including SLC1A5, SLC7A5, SLC7A11, and SLC6A14 (Bhutia et al., 2015), MCTs and GLUTs have been found to be highly expressed in tumor tissues. This phenomenon suggests that there is great potential for the future development of new tumor-imaging probes and tumor-specific delivery of appropriately designed chemotherapeutic agents. For example, the use of BPA-based boron neutron capture therapy significantly enhances the efficiency of SLC7A5 (LAT-1)-mediated uptake of p-boronophenylalanine into cancer cells (Seneviratne et al., 2022).

In addition to developing SLC-targeted synergistic anti-tumor therapies based on the unique metabolic demands of tumors, certain drugs themselves directly interact with members of the SLC protein family. The antiviral drug valacyclovir and the antibacterial drug cefadroxil are substrates for the peptide transporters PEPT1 (SLC15A1) and PEPT2 (SLC15A2) (Ganapathy et al., 1998; Yang and Smith, 2013; Fernandez-Prado et al., 2024). The hepatitis D drug bulevirtide inhibits NTCP transport activity, thereby exerting antiviral effects (Liu et al., 2024). OCT1 (SLC22A1) is the transporter for metformin, responsible for transporting metformin into hepatocytes to exert its glucose-lowering effects (Mofo Mato et al., 2018). These direct connections between drugs and SLC transporters suggest that researchers can leverage the tissue-specific distribution of SLC transporters to design drugs targeting these transporters, thereby enhancing efficacy and selectivity while reducing toxicity. Additionally, drug structures can be optimized, prodrugs can be developed, or nano-delivery systems can be utilized to increase the affinity of drugs for SLC transporters, improving drug delivery efficacy.

### 4.3 Challenges and advances in SLC protein research

Currently, the vast majority of SLC protein family members in clinical trials have not been successfully explored. There are still some technical challenges in the study of SLC proteins. Three-dimensional structural analysis of SLC proteins is one of the most effective ways to explore their function and transport mechanisms. Benefiting from advancements in cryo-electron microscopy technologies for determining the structures of membrane proteins (Schlessinger et al., 2023), the number of experimentally determined SLC protein structures has surged in recent years. Although approximately 92% of known drug target structures have been deposited in protein databases; most SLC proteins have not been crystallized due to sample preparation and crystallization challenges. This limits computer-aided drug discovery. Additionally, SLC proteins localized in intracellular compartments may not be ideal targets for drug therapies using current drug delivery approaches. Beyond the significant challenges in elucidating the structures of SLC proteins, many orphan transporters lack identified substrates, numerous well-characterized SLC transporters have no known chemical modulators, and a variety of disease-associated mutations in SLCs remain uncharacterized (Lin et al., 2015b).

The development of gene editing technology and the construction of animal models provide powerful means for the treatment of metabolic diseases, and the full use of these technologies can effectively confirm the feasibility of potential therapeutic targets. In recent years, researchers have used the CRISPR/Cas9 system, adeno-associated viruses and other efficient gene editing tools to construct a large number of SLC knockout/knockdown or SLC-overexpressing animal models for screening disease risk sites and drug targets, which has greatly promoted the use of SLC transporters as therapeutic targets for metabolic diseases. At the same time, gene editing technology also provides hope for the treatment of genetic metabolic diseases caused by SLC family protein-encoding gene mutations. The gene editor delivered by the adeno-associated virus carrier can accurately replace the mutant base pairs of cells and achieve the therapeutic purpose of transforming the mutant cells into normal cells.

However, previous studies have focused on single proteins, ignoring the similarities and correlations between SLC transporter members. Therefore, future studies need to build animal models of two or more SLC gene cointerventions to reveal the interactions between SLC transporters, especially those SLC transporters that share the same transport substrate.

## 5 Conclusion

The global prevalence of metabolic diseases, such as obesity, diabetes, and NAFLD, is rapidly increasing, posing significant challenges to public health. SLC transporters, the second largest family of membrane proteins in the human genome, are crucial for maintaining energy metabolism, homeostasis, and cellular functions. This review highlights the roles of various SLC families in metabolic diseases, their tissue distribution, and their potential as therapeutic targets. While fewer than 20 SLC-targeted drugs have been successfully marketed, advances in artificial intelligence and gene editing technologies are enabling deeper insights into the structure and function of SLC proteins. Targeting SLC transporters for the treatment of metabolic diseases holds great promise for future therapeutic development.

## Glossary

T2D: Type 2 diabetes mellitus
NAFLD: Nonalcoholic fatty liver disease
HGNC: The Genome Organization Gene Nomenclature Committee
SGLT: Sodium-dependent glucose transporter
GLUT: Glucose transporter
FATPs: Fatty acid transport proteins
NASH: Nonalcoholic steatohepatitis
SNPs: single nucleotide polymorphisms
ASBT: Sodium-dependent bile acid transporter
FXR: Farnesol X receptor
NTCP: Na^+^ -taurocholate co-transport polypeptide
Tgr5: G protein-coupled bile acid receptor
BAs: Bile acids
BSEP: Bile salt export pump
SOAT: Sodium ion-dependent organic anion transporter
NPT: Na^+^ dependent phosphate transporter
VGLUT: vesicular glutamate transporters
VNUT: vesicular nucleotide transporter
OAT: organic anion transporter
OCT: organic cation transporter
OCTN: organic cation/carnitine transporter
SO42-: Inorganic anion sulfate
NAS1: The Na^+^ -sulfate cotransporter
SAT1: Sulfate anion transporter
NAS: Sodium sulfate cotransporters
NADC: Sodium-dependent dicarboxylate transporters
MCT: Monocarboxylate transporter protein
ZIP: Zrt-and Irt-like protein family
ZnT: Zinc transporter ZnT family
T1D: Type 1 diabetes mellitus
TCA: Tricarboxylic acid cycle
UCP: Uncoupling protein
SDCT: High-affinity sodium-dependent dicarboxylate co-transporter
PPARγ: Peroxisome proliferator-activated receptor γ
CACT: Carnitine/acylcarnitine translocase
LC-FAOD: long-chain fatty acid oxidation disorders
IBS: Irritable bowel syndrome
PBC: Primary biliary cholangitis
NTIS: Non-thyroidal illness syndrome
TNZD: Transient neonatal zinc deficiency
